# A new species of *Inosperma*, and first record of *I*. *afromelliolens* (*Inocybaceae*, Fungi) from West Africa

**DOI:** 10.1371/journal.pone.0290894

**Published:** 2023-10-18

**Authors:** Hyppolite L. Aïgnon, Yu-Guang Fan, André De Kesel, Mohammad Bahram, Martin Ryberg, Nourou S. Yorou

**Affiliations:** 1 Research Unit Tropical Mycology and Plants-Soil Fungi Interactions, Faculty of Agronomy, University of Parakou, Parakou, Benin; 2 Key Laboratory of Tropical Translational Medicine of Ministry of Education Tropical Environment and Health Laboratory, School of Pharmacy, Hainan Medical University, Haikou, China; 3 Meise Botanic Garden, Meise, Belgium; 4 Department of Ecology, Swedish University of Agricultural Sciences, Uppsala, Sweden; 5 Institute of Ecology and Earth Sciences, University of Tartu, Tartu, Estonia; 6 Systematic Biology Program, Department of Organismal Biology, Uppsala University, Uppsala, Sweden; ICAR-Directorate of Mushroom Research, INDIA

## Abstract

Here, we present the newly identified *Inosperma macrocarpa* and the first record of *I*. *afromelliolens* from West Africa. *Inosperma macrocarpa* is nested in an Old World Tropical clade, based on a molecular phylogeny inferred from the sequences of *ITS*, *LSU*, *RPB2*, and *TEF1*. Complete descriptions and illustrations, including photographs and line drawings, of the new species are presented. Morphological and molecular analyses based on collections from Benin confirmed the presence of *I*. *afromelliolens* in West Africa. Toxicity analysis showed that neither species contained muscarine, which further supports the hypothesis that the ability to produce muscarine is a derived trait of *Inosperma*.

## Introduction

*Inocybaceae* Jülich (*Basidiomycota*, *Agaricales*) is one of the most taxonomically diverse families of *Agaricales* with seven genera, including *Inosperma* (Kühner) Matheny & Esteve-Rav. [1]. Members of *Inosperma* are morphologically characterized by basidiomes of small to medium size; occasional distinctive odors such as fruity, honey, fishy, or pleasant; rimose or squamulose pileus, smooth stipe, with a bulbous base and/or bruising reaction; smooth, ellipsoid, or phaseoliform to subglobose basidiospores; thin-walled cheilocystidia; and a lack of pleurocystidia [1, 2].

This genus is monophyletic, and four distinct clades have been recognized within it: two distinct tropical old-world clades (1 and 2) [1, 3–5], the Maculata clade [6], and *Inosperma* sect. *Inosperma* [4]. Most species of Old World Tropical clade 2 are primarily from tropical Asia [5], whereas taxa of Old World Tropical clade 1 are mainly found in tropical Africa [1, 4]. This genus includes a few toxic species [7] with high levels of muscarine, including *Inosperma erubescens* (A. Blytt) Matheny & Esteve-Rav, the species in *Inocybaceae* responsible for most mushroom poisoning [5, 8, 9]. Species of *Inosperma* have been consistently reported in mushroom poisoning incidents in tropical Asia [10–12]; however, the toxicity of members of this genus in Africa remains poorly studied. Thus, documenting the diversity of *Inosperma* in tropical Africa and their toxicity can help avoid food poisoning due to mushroom consumption.

Here, based on the morphological characteristics and multigene molecular analysis using *ITS*, *LSU*, *RPB2*, and *TEF1* sequence data, we describe *Inosperma macrocarpa* sp. nov. and report the first West African record of *I*. *afromelliolens*. The toxicity of both taxa was studied by analyzing their muscarine content.

## Material and methods

### Study area and specimen sampling

The specimens were collected between 1997 and 2022 in Benin, woodlands dominated by *Isoberlinia doka* Craib & Stapf and/or *I*. *tomentosa* (Harms) Craib & Stapf, or gallery forests dominated by *Uapaca guineensis* Müll. Arg and/or *Berlinia grandiflora (*Vahl) Hutch. & Dalziel, in Okpara Forest (9.270131°N, 2.715440°E), N’dali Forest Reserve (9.758857°N, 2.696819°E), Koussoucoingou gallery forest (10.173066°N, 1.196233°E), Forest reserve of Gbadji (7.952167°N, 1.967867°E) and Forest reserve of Wari Maro (9.164733°N, 2.159917°E). No specific permits were required for mushroom sample collection for the Beninese researcher in the forest reserve.

The fresh basidiomata were dried using an electric Stöckli Dörrex dryer for 24 hours at 45°C. Most specimens studied, including the holotype of the newly described species, were deposited in the Mycological Herbarium of Parakou University, Benin Republic (UNIPAR), and additional specimens of *I*. *macrocarpa* (vouchers ADK2166 and ADK2618, leg. A. De Kesel) were deposited in the Meise Botanic Garden Herbarium (BR).

### Morphological analysis

Specimens were photographed in the field using a digital camera (Sony ILCE 7RM3), and the colors were described based on Kornerup and Wanscher [13]. Fine sections from the dried basidiomata were rehydrated and examined in 3% KOH and Congo Red for microscopic investigation. The microscopic characteristics were drawn using a drawing tube attached to a Leica DM2700 light microscope.

Microscopic characteristics were drawn at 1000× magnification and 120 spores in three collection samples for each species were measured. We measured the length (L) and width (W) of basidiospores and calculated the ratio Q = L/W. The spore dimensions are given as (a–)b–c–d(–e), where (a) represents the extreme values at the < 5th percentile, the range b–d refers to the minimum of 90% of the measured values, (c) represents the average value, and (e) represents the extreme values at the < 95th percentile. Measurements of basidiospores and basidia excluded the apiculi and sterigmata.

### DNA extraction and PCR and sequencing

Genomic DNA was extracted from dried specimens using a NuClean Plant Genomic DNA kit (ComWin Biotech, Beijing, China). The full *ITS* region and parts of *LSU*, *RPB2*, and *TEF1* were amplified. We produced amplicons using the primers ITS1F/ITS4 for *ITS* [14, 15], LR0R/LR7 for *LSU* [16–18], bRPB2-6F/bRPB2-7.1R for *RPB2* [18], and EF1-983F/EF1-1576R for *TEF1* [20]. All PCR products were sent to the Beijing Genomics Institute (Beijing, China) for purification and sequencing using the same primers as those used for PCR.

### Sequence alignment and phylogenetic analysis

All new sequences in this study were prepared and compared with closely related *Inosperma* sequences retrieved from GenBank [21]. All *Inosperma* species described from Africa were included in the phylogenetic analysis. Sequences from other genera of *Inocybaceae* were added, based on Matheny et al. [1] and Aïgnon et al. [4] (Table 1). Sequences of different regions (*ITS*, *LSU*, *RPB2*, and *TEF1*) were aligned separately using MAFFT v7.511 [22], and a final concatenated dataset of *ITS*, *LSU*, *RPB2*, and *TEF1* was generated using Geneious 7.0.2 [23].

**Table 1:** Taxon sampling information and DNA sequences used for phylogenetic analysis.

Species	Voucher	Country	*ITS*	*LSU*	*RPB2*	*TEF1*	References
*Auritella brunnescens* Matheny & Bougher	PBM3174	Australia	KJ702344	JQ313571	KJ702349	MK426176	[24]
*Auritella dolichocystis* Matheny, Trappe & Bougher	Trappe 24844	New South Wales	–	AY380371	AY337371	–	[19]
*Auritella fulvella* Matheny & Bougher	AQ669485	Australia	KJ702355	KJ702353	KJ702357	MK426178	[24]
*Auritella hispida* Matheny & T.W. Henkel	TH1009, TH10379	Cameroon	KT378203	KT378208	KT378215	MK426179	[24]
*Auritella serpentinocystis* Matheny, Trappe &Bougher ex Matheny & Bougher	PBM3188	Australia	KJ729858	JQ313559	KJ756402	MK426181	[24]
*Auritella spiculosa* Matheny & T.W. Henkel	TH9866	Cameroon	KT378204	KT378206	KT378214	MK426182	[24]
*Inocybe beninensis* Aïgnon, Yorou & Ryberg	HLA0390	Benin	MN096196	MN097888	NA	–	[25]
*Inocybe flavoalbida* Matheny & Bougher	PBM3768	Australia	KJ729873	KJ729901	MK426183	MK426183	[1]
*Inocybe fuscicothurnata* Grund & D.E. Stuntz	PBM3980	USA	MF487844	KY990485	MK426184	MK426184	[1]
*Inocybe humidicola* Matheny & Bougher	PBM3719	Australia	KP171126	KJ801181	MK426185	MK426185	[26]
*Inocybe hydrocybiformis* (Corner & E. Horak) Garrido	ZT10077	Thailand	GQ893016	GQ892971	–	–	Unpublished
*Inocybe lanuginosa* (Bull.) P. Kumm.	PBM956, PBM3023	USA	HQ232480	KP170923	MK426186	MK426186	[26]
*Inocybe lasseroides* (E. Horak) Garrido	PBM3749	Australia	KP171145	KP170924	MK426187	MK426187	[27]
*Inocybe luteifolia* (E. Horak) Garrido 1988	PBM2642, AHS6557	USA	FJ436331	EU307814	MK426188	MK426188	[1]
*Inocybe magnifolia* Matheny, Aime & T.W. Henkel	MCA2441	Guyana	JN642228	JN642244	MK426189	MK426189	[1, 28]
*Inocybe melanopoda* D.E. Stuntz	PBM3975	USA	–	MH220276	MK426190	MK426190	[1]
*Inocybe pallidicremea* Grund & D.E. Stuntz	PBM2039, PBM2744	USA	KY990553	AY380385	MK426191	MK426191	[1, 19]
*Inocybe persicinipes* Matheny & Bougher	PBM2197	USA	KF977215	EU600837	MK426192	MK426192	[1, 29]
*Inocybe pileosulcata* E. Horak, Matheny & Desjardin	TBGT10742	India	KP308810	KP170979	MK426193	MK426193	[1, 30]
*Inocybe pluvialis* Matheny, Bougher & G.M. Gates	PBM3228	Australia	KF871777	KF853401	MK426194	MK426194	[1, 26]
*Inocybe roseifolia* Murrill	CO5576	USA	MH578026	MK421968	MK426195	MK426195	[1]
*Inocybe rufobadia* Matheny & Bougher	NLB885	Australia	KF977213	KF915290	MK426196	MK426196	[1, 26]
*Inocybe serrata* Cleland	PBM3235	Australia	KP636810	KP171012	MK426197	MK426197	[1, 26]
*Inocybe spadicea* Matheny & Bougher	PBM2203	Australia	KP636866	EU600865	–	MK426198	[1, 26, 29]
*Inocybe stellata* E. Horak, Matheny & Desjardin	ECV3651	Thailand	GQ893007	GQ892962	KM656105	–	[27]
*Inocybe subexilis* (Peck) Sacc.	PBM2620, ACAD11680	Canada	MH578001	EU307845	MK426199	MK426199	[1, 31]
*Inocybe thailandica* E. Horak, Matheny & Desjardin	DED8049	Thailand	GQ893013	GQ892968	MK426200	MK426200	[1, 27, 29]
*Inocybe tubarioides* G.F. Atk.	PBM2550	USA	EU439453	AY732211	MK426201	MK426201	[1, 32]
*Inosperma adaequatum* (Britzelm.) Matheny & Esteve-Rav.	JV 16501F, JV11290F	Finland	JQ801381	JQ815407	AY333771	MK426202	[1]
*Inosperma africanum* Aïgnon, Yorou & Ryberg	MR00387	Togo	MN096189	MN097881	MT770739	–	[4]
*Inosperma afromelliolens* Eyssart. & Buyck	PC 96013	Zambia	JQ801383	JQ815408	EU600882	–	[29]
*Inosperma afromelliolens* Eyssart. & Buyck	**HLA0469**	**Benin**	**MT534294**	**MT536757**	–	–	This study
*Inosperma afromelliolens* Eyssart. & Buyck	**HLA0407**	**Benin**	**MT534296**	**MT560736**	–	–	
*Inosperma afromelliolens* Eyssart. & Buyck	**HLA0405**	**Benin**	**MT534292**	**MT560737**	–	–	
*Inosperma afromelliolens* Eyssart. & Buyck	**HLA0754**	**Benin**	**OQ300372**	**OQ300369**	**OQ435246**	**OQ441164**	
*Inosperma akirnum* (K.P.D. Latha & Manimohan) Matheny & Esteve-Rav.	CAL 1358	India	–	NG_057279	KY553236	–	[30]
*Inosperma apiosmotum* (Grund& D.E. Stuntz) Matheny & Esteve-Rav.	AU10560, TENN:062779	Canada, USA	HQ201336	JN975022	JQ846463	–	[33]
*Inosperma bicoloratum* (E. Horak, Matheny & Desjardin) Matheny & Esteve-Rav.	ZT12187	Malaysia	GQ892984	GQ892938	JQ846464	–	[27]
*Inosperma boeticum* Eyssart. & Buyck	PC96082	Zambia	JQ801412	JN975027	–	–	[33]
*Inosperma bongardii* (Weinm.) Matheny & Esteve-Rav.	EL9406	Sweden	FN550943	FN550943	–	–	Unpublished
*Inosperma bulbomarginatum* Aïgnon, Yorou & Ryberg	MR00357	Benin	MN096190	MN097882	MN200775	–	[4]
*Inosperma calamistratoides* (E. Horak) Matheny & Esteve-Rav.	PBM3384	Australia	–	JQ815415	KJ729949	–	Unpublished
*Inosperma calamistratum* (Fr.) Matheny & Esteve-Rav.	PBM1105	USA	JQ801386	JQ815409	JQ846466	MK426203	[34]
*Inosperma carnosibulbosum* (C.K. Pradeep & Matheny) Matheny & Esteve-Rav.	TBGT:12047	India	KT329448	KT329454	KT32944	MK426205	[34]
*Inosperma cervicolor* (Pers.) Matheny & Esteve-Rav.	SJ04024, TURA:4761	Sweden, Finland	AM882939	AM882939	JQ846474	–	[35]
*Inosperma cookei* (Bres.) Matheny & Esteve-Rav.	EL70A03	Sweden	AM882953	AM882953	–	–	[35]
*Inosperma cyanotrichium* (Matheny, Bougher& G.M. Gates) Matheny & Esteve-Rav	TENN:065729	Australia	–	JQ815418	KJ729948	–	Unpublished
*Inosperma fastigiellum* (G.F. Atk.) Matheny & Esteve-Rav	JRH 408	USA		AY380374	AY333770		[19]
*Inosperma flavobrunneum* Aïgnon, Yorou & Ryberg	**HLA0746**	**Benin**	**OQ446450**	**OQ293897**	**OQ435247**	**OQ441163**	This study
*Inosperma flavobrunneum* Aïgnon, Yorou & Ryberg	**HLA0367**	**Benin**	**MN096199**	**MT536754**	**–**	**–**	
*Inosperma flavobrunneum* Aïgnon, Yorou & Ryberg	**HLA0759**	**Benin**	**OQ436032**	**OQ293896**	**OQ435248**	**OQ441165**	
*Inosperma fulvum* (Bon) Matheny & Esteve-Rav.	TURA1812	Finland	JQ408763	JQ319694	JQ846484	–	Unpublished
*Inosperma geraniodorum* (J. Favre) Matheny & Esteve-Rav.	EL10606	Sweden	FN550945	FN550945	–	–	Unpublished
*Inosperma gregarium* (K.P.D. Latha & Manimohan) Matheny & Esteve-Rav.	CAL 1309	India	KX852305	KX852306	KX852307	–	[19]
*Inosperma hainanense* Y.G. Fan, L.S. Deng, W.J. Yu & N.K. Zeng	Zeng4937	China	MZ374070	MZ374761	MZ388104	MZ388107	[5]
*Inosperma lanatodiscum* (Kauffman) Matheny & Esteve-Rav.	PBM2451	USA	JQ408759	JQ319688	JQ846483	–	[36]
*Inosperma longisporum* S.N. Li, Y.G. Fan & Z.H. Chen	MHHNU 33070	China	OP135504	OP135495	OP161564	–	[12]
***Inosperma macrocarpa* Aïgnon & Yorou**	**HLA0787**	**Benin**	**OQ300390**	**OQ286290**	**OQ427873**	**OQ441166**	**This study**
***Inosperma macrocarpa* Aïgnon & Yorou**	**HLA0788**	**Benin**	**OQ300391**	**OQ286291**	**OQ435242**	**OQ441167**	
***Inosperma macrocarpa* Aïgnon & Yorou**	**HLA0790**	**Benin**	**OQ300392**	**OQ286292**	**OQ435243**	**OQ441168**	
***Inosperma macrocarpa* Aïgnon & Yorou**	**HLA0792**	**Benin**	**OQ300393**	**OQ286293**	**OQ435245**	**OQ441170**	
***Inosperma macrocarpa* Aïgnon & Yorou**	**HLA0791**	**Benin**	**OQ300373**	**OQ300370**	**OQ435244**	**OQ441169**	
*Inosperma maculatum* (Boud.) Matheny & Esteve-Rav.	MR00020, TENN:071817	Sweden/USA	AM882958	MT228862	MH577496	–	[35]
*Inosperma maximum* (A.H. Sm.) Matheny & Esteve-Rav.	PBM 2222,UBC F33244	USA/Canada	MG953983	EU569854	–	–	[29]
*Inosperma misakaense* (Matheny & Watling) Matheny &Esteve-Rav.	96234 (PC)	Zambia	JQ801409	EU569874	EU569873	MK426206	[34]
*Inosperma muscarium* Y.G. Fan, L.S. Deng, W.J. Yu & N.K. Zeng	FYG6091	China	MZ373982	MZ373991	MZ388093	MZ388100	[5]
*Inosperma mutatum* (Peck) Matheny & Esteve-Rav.	PBM4108, PBM2953	USA	MG773837	JQ994476	JQ846488	MK426207	[1]
*Inosperma neobrunnescens* (Grund & D.E. Stuntz) Matheny &Esteve-Rav.	PBM 2452	USA	–	EU569868	EU569867	–	[29]
*Inosperma nivalellum* S.N. Li, Y.G. Fan & Z.H. Chen	MHHNU 31689	China	OP135502	OP134006	OP161556	–	[12]
*Inosperma quietiodor* (Bon) Matheny & Esteve-Rav.	PAM01091310	France	FJ936168	FJ936168	–	–	[6]
*Inosperma rhodiolum* (Bres.) Matheny & Esteve-Rav.	PAM00090117	France	FJ904176	FJ904176	–	–	[6]
*Inosperma rimosoides* (Peck) Matheny & Esteve-Rav.	PBM 2459, PBM3311	USA	JQ801414	JQ815426	DQ385884	DQ435790	[36]
*Inosperma rubricosum* (Matheny & Bougher) Matheny & Esteve-Rav.	PBM3784	Australia	–	NG_057260	KM406230	–	[27]
*Inosperma submaculatum* Eyssart. & Buyck	PC96073	Zambia	JQ801417	EU600870	EU600869	–	[29]
*Inosperma shawarense* (A. Naseer & A.N. Khalid) Aïgnon & Naseer	FLAS-FS9456	Pakistan	KY616965	KY616966	–	–	[37]
*Inosperma* sp.	CO4253	USA	MH578032	–	MH577500	–	Unpublished
*Inosperma* sp.	BB3233	Zambia	JQ801415	EU600885	–	–	[29]
*Inosperma* sp.	D78	Thailand	MW538592	MW538617	MW512902	–	[11]
*Inosperma* sp.	D80	Thailand	MW538594	MW538618	MW512903	–	
*Inosperma* sp.	D83	Thailand	MW538597	MW538619	MW512904	–	
*Inosperma* sp.	D85	Thailand	MW538599	MW538620	MW512905	–	
*Inosperma* sp.	D86	Thailand	MW538600	MW538621	MW512906	–	
*Inosperma* sp.	D89	Thailand	MW538601	MW538622	MW512907	–	
*Inosperma* sp.	D152	Thailand	MW538604	MW538623	MW512908	–	
*Inosperma* sp.	G1842	Zambia	–	MK278245	–	–	Unpublished
*Inosperma* sp.	PBM2355	Norway	–	AY380402	AY333768	–	[19]
*Inosperma* sp.	PBM2871	USA	HQ201348	HQ201348	JQ846475	–	Unpublished
*Inosperma* sp.	PBM3406	Australia	–	JQ815431	JQ846498	–	Unpublished
*Inosperma* sp.	SAT0427406	USA	JQ801411	JN975025	JQ846489	–	Unpublished
*Inosperma* sp.	TJB10045	Thailand	KT600658	KT600659	KT600660	–	[34]
*Inosperma* sp.	TR220_06	Papua New Guinea	JQ801416	JN975017	JQ846496	–	[33]
*Inosperma sphaerobulbosum* S.N. Li, Y.G. Fan & Z.H. Chen	MHHNU 32266	China	OP135501	OP389205	OP161559	–	[12]
*Inosperma squamulosobrunneum* Y.G. Fan, L.S. Deng, W.J. Yu & L.Y. Liu	MHHNU 32359	China	OP135499	OP134000	OP161562	–	[12]
*Inosperma subsphaerosporum* Y.G. Fan, L.S. Deng, W.J. Yu & L.Y. Liu	FHMU3155	China	MW403827	MW397173	MW404239	–	[5]
*Inosperma vinaceobrunneum* (Matheny, Ovrebo & Kudzma) Haelew.	TENN:062709, PBM 2951	USA	FJ601813	NG_067775	JQ846478	–	[38]
*Inosperma viridipes* (Matheny, Bougher & G.M. Gates) Matheny & Esteve-Rav.	PBM3767	Australia	NR_153168	KP171094	KM656138	–	[27]
*Inosperma virosum* (K.B. Vrinda, C.K. Pradeep, A.V. Joseph & T.K. Abraham ex C.K. Pradeep, K.B. Vrinda& Matheny) Matheny & Esteve-Rav.	TBGT:753	India	KT329452	KT329458	KT329446	MK426208	[34]
*Inosperma zonativeliferum* Y.G. Fan, H.J. Li, F. Xu, L.S. Deng & W.J. Yu	FYG6441	China	OL850878	OM845772	ON075044	–	[7]
*Mallocybe africana* Aïgnon, Yorou & Ryberg	MR00385	Togo	MN096194	MR00385	MT465593	–	[39]
*Mallocybe fibrillosa* (Peck) Matheny & Esteve-Rav.	LVK22085	USA	OP917925	OP918013	–	–	Unpublished
*Mallocybe fulvoumbonata* (Murrill) Matheny & Esteve-Rav.	TENN:075560	USA	MZ404931	MZ375433	MZ405011	–	Unpublished
*Mallocybe isabellina* (Matheny & Bougher) Matheny & Esteve-Rav.	PERTH:08073287	Australia	KP171142	KP170921	KJ811587	–	Unpublished
*Mallocybe latispora* (Bon) Matheny & Esteve-Rav.	TENN:063759	Finland	MN178505	MN178531	MN203522	–	Unpublished
*Mallocybe leucoblema* (Kühner) Matheny & Esteve-Rav.	TENN:062549	USA	HQ232481	MN178534	–	–	Unpublished
*Mallocybe malenconii* (R. Heim) Matheny & Esteve-Rav.	JV5824F	Finland	–	MN178538	–	–	Unpublished
*Mallocybe myriadophylla* (Vauras & E. Larss.) Matheny & Esteve-Rav.	JV19652F	Finland	DQ221106	AY700196	AY803751	DQ435791	[40]
*Mallocybe pyrrhopoda* (Matheny & Bougher) Matheny & Esteve-Rav.	PERTH:08362963	Australia	–	KP170985	–	–	Unpublished
*Mallocybe* sp.	PBM 2350	USA	–	EU600834	EU600833	–	[29]
*Mallocybe subdecurrens* (Ellis & Everh.) Matheny & Esteve-Rav.	REH10168	USA	MH024850	MH024886	MH577503	MK426209	[1]
*Mallocybe subflavospora* (Matheny & Bougher) Matheny & Esteve-Rav.	PERTH:08320861	Australia	MN178515	KP171074	KM656118	–	[27]
*Mallocybe subtilior* (Matheny & Bougher) Matheny & Esteve-Rav.	PERTH:08095388	Australia	KP641628	KP171082	–	–	Unpublished
*Mallocybe terrigena* (Fr.) Matheny, Vizzini & Esteve-Rav.	EL11704, JV 16431	Sweden	AM882864	AY380401	AY333309	–	[19, 41]
*Mallocybe tomentosula* Matheny & Esteve-Rav.	PBM4138	USA	MG773814	MK421969	MH577506	MK426210	[1]
*Mallocybe unicolor* (Peck) Matheny & Esteve-Rav.	PBM 1481, PBM2589	USA	–	AY380403	AY337409	MK426211	[19]
*Nothocybe distincta* (K.P.D. Latha & Manim.) Matheny & K.P.D. Latha	ZT9250	India	–	EU604546	EU600904	MK426212	[1, 29]
*Pseudosperma bulbosissimum* (Kühner) Matheny & Esteve-Rav.	DBG:19916	USA	MH024849	MH024885	MH249788	MK426213	[1]
*Pseudosperma cercocarpi* (Kropp, Matheny & L.J. Hutchison) Matheny & Esteve-Rav.	BK20069806	USA	MK421964	EU600890	EU600889	MK426214	[1]
*Pseudosperma lepidotellum* (Matheny & Aime) Matheny & Esteve-Rav.	TENN066442	Guyana	JN642233	NG_042597	MH577508	–	[28]
*Pseudosperma luteobrunneum* (K.P.D. Latha & Manim.) Matheny & Esteve-Rav.	CAL 1260	India	NR_153171	NG_057275	KX073588	–	[42]
*Pseudosperma notodryinum* (Singer, I.J.A. Aguiar & Ivory) Matheny & Esteve-Rav.	CSU<USA-OK>:0125	USA	NR_164070	MK421970	MH577509	MK426216	[1]
*Pseudosperma pluviorum* (Matheny & Bougher) Matheny & Esteve-Rav.	BRI:AQ794010, PERTH:08556466	Australia	–	NG_057259	KM406221	MK426217	[27]
*Pseudosperma* sp.	PBM3751	Australia	KP636851	KP171053	KM555145	–	[34]
*Pseudosperma* sp.	TR194-02 (M)	Papua New Guinea	JQ408793	JN975032	JQ421080	–	[33]
*Pseudosperma spurium* (Jacobsson & E. Larss.) Matheny & Esteve-Rav.	BK180809723	USA	JQ408794	EU600868	MK426219	MK426219	[1]
*Tubariomyces hygrophoroides* Esteve-Rav., P.-A. Moreau & C.E. Hermos.	P05112008	France	GU907097	GU907094	GU907090	–	[34]
*Tubariomyces inexpectatus* (M. Villarreal, Esteve-Rav., Heykoop & E. Horak) Esteve-Rav. & Matheny	AH25500, AH20390	Spain	GU907095	EU569855	GU907088	–	[29, 43]
*Tubariomyces similis* Della Magg., Tolaini & Vizzini	RFS0805	Spain	GU907096	GU907092	GU907089	–	[43]

The dataset was partitioned into *ITS* and *LSU*, and the different codon positions of *RPB2* and *TEF1* were partitioned separately, and their introns and separate models of DNA substitution were applied to the first, second, and third codon positions of the protein-coding genes. For phylogenetic analysis, substitution models and the best partitioning schemes were determined for Maximum Likelihood (ML). Substitution models for each locus were determined based on the AICc model selection criterion implemented in PartitionFinder [44].

ML analysis was performed using IQ-TREE v2.2.0 [45]. Ultrafast bootstrapping (UFBoot) was performed using 1000 replicates [46]. Sequences of *Inocybe* (Fr.) Fr., *Nothocybe* Matheny & K.P.D. Latha, and *Pseudosperma* Matheny & Esteve-Rav. were used for rooting [47].

### Nomenclature

The electronic version of this article in the portable document format (PDF) in a publication with an ISSN or ISBN will represent published work according to the International Code of Nomenclature for algae, fungi, and plants. Hence, the new names contained in the electronic publication of a PLOS ONE article are effectively published under that Code from the electronic edition alone; therefore, no printed copies need to be provided.

The new names contained in this work have been submitted to MycoBank, where they will be made available to the Global Names Index. The unique MycoBank number can be resolved, and the associated information can be viewed through any standard web browser by appending the MycoBank number contained in this publication to the prefix http://www.mycobank.org/MB/. The online version of this work was archived and made available in the following digital repositories: PubMed Central and LOCKSS.

### Muscarine detection

Dried samples were ground into a fine powder, 2.5–26 mg of each specimen was weighed and placed into a 5 mL centrifugation tube with 2 mL of methanol-water (5:95, v/v). The mixture was vortexed for 30 min and ultrasonically extracted for another 30 min. After 5 min of centrifugation at 10000 rpm, the total supernatant was collected, filtrated using a 0.22 μm organic filter membrane, and mixed with acetonitrile-water (7:3, v/v) to a final volume of 1 mL for UPLC-MS/MS analysis. *Lentinula edodes* (Berk.) Pegler was used as a blank sample.

The UPLC-MS/MS analysis was performed using an ABSCIEX Exion UPLC system coupled to an ABSCIEX Triple Quad 6500+ system (ABSCIEX). Chromatographic separation was achieved on an ACQUITY UPLC Amide column (2.1 × 100 mm, 1.7 μm, Waters, USA). Aqueous solutions of 0.05% formic acid (A) and acetonitrile (B) were used as the mobile phase solvent flowing at 0.3 mL/min. The column was eluted with 70–10% B for 3 min, followed by 10% B for 0.5 min and then by 10–70% B for 0.5 min, and 70% B for 2 min. The analytical column was set at 40°C and the injection volume was 2.0 μL [48]. The muscarine content was estimated in the UPLC-MS/MS by using standard muscarine (Sigma-Aldrich, Chemical purity ≥ 98%), and was calculated with an external standard method based on respective calibration curves.

The protonated molecular ion ([M+H]^+^) of 174.2 was chosen as the parent ion, as well as two daughter ions at 57.0 and 97.0, which were used for qualitative and quantitative detection, respectively. The MS/MS conditions were as follows: ion source, electrospray ionization; curtain gas, 20 psi; collision gas, 8 psi; ionspray voltage, 5500 V; ion source temperature, 500°C; ion source gas, 1, 50 psi; ion source gas, 2, 50 psi. Product ion confirmation (PIC) was set as follows: scan function, negative ion scanning; scan mode: multiple reaction monitoring; PIC duration for 0.21 s; collision energy at 27 V. The Analyst software (version 1.6) was used for data acquisition and processing.

## Results

### Phylogenetic analysis

**Fig 1:**
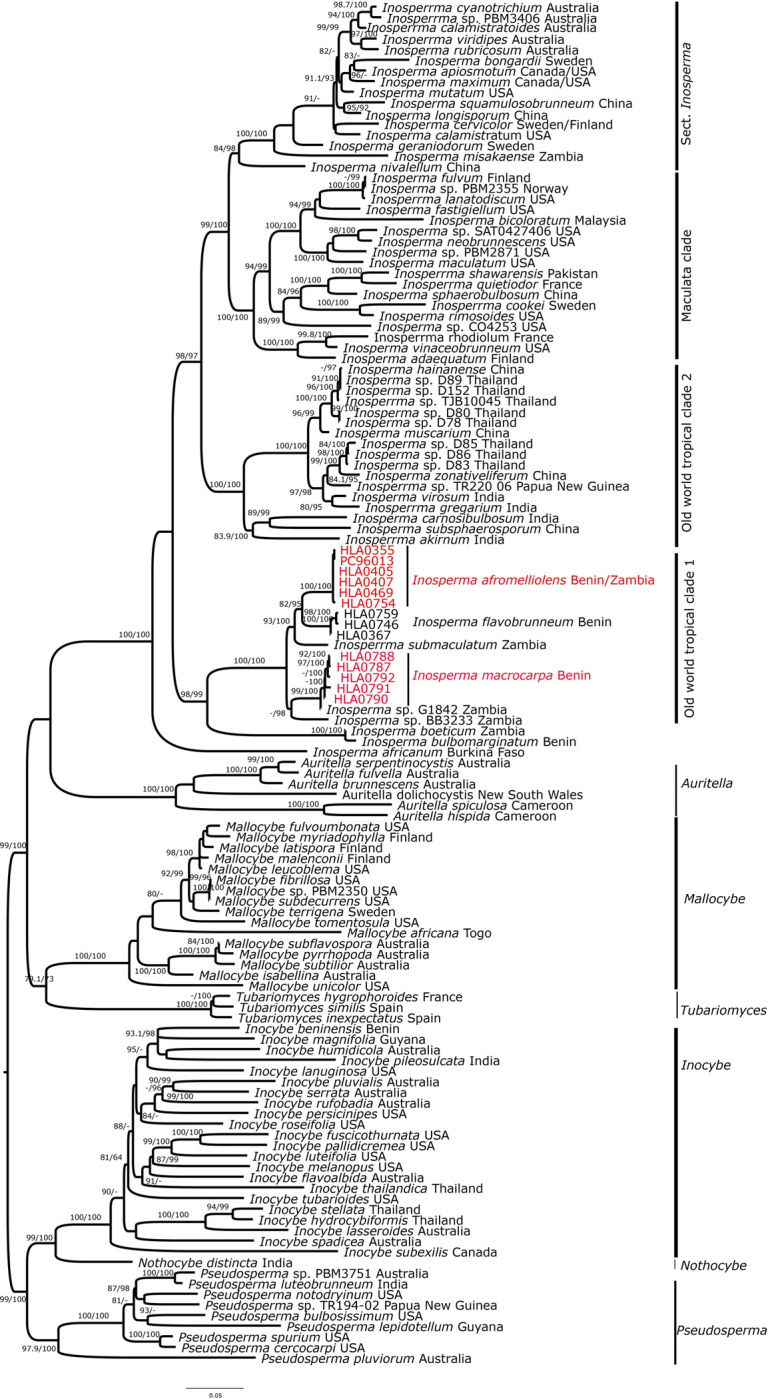
ML tree of *ITS*, *LSU*, *RPB2*, and *TEF1* sequences showing the placement of *Inosperma macrocarpa*. Values above or below branches indicate bootstrap proportions SH-aLRT support ≥ 80% / ultrafast bootstrap support ≥ 95%. Origin of species is given after the name of each taxon. The new species and the new records are in red.

Approximately 45 new sequences were submitted to GenBank. The sequences used for the phylogenetic analyses are presented in Table 1. The *ITS* locus was present in 110 taxa and the alignment had 909 sites; the *LSU* locus was present in 127 taxa and the alignment had 1470 sites; the *RPB2* locus was present in 105 taxa and the alignment had 778 sites; and the *TEF1* locus was present in 51 taxa and the alignment had 1113 sites. Multigene molecular analysis of *ITS*, *LSU*,, and *TEF1* sequence data grouped the newly sampled specimens into two separate clades, each with short branches within the clade relative to the branch leading to the clade (Fig 1). One clade also included a specimen of *Inosperma afromelliolens* and we concluded that our samples were conspecific to that specimen. The other clade did not include any specimens annotated with a formal name and was determined to be a previously undescribed species (*Inosperma macrocarpa*; Fig 1).

Phylogenetically, *I*. *macrocarpa* is nested in Old World Tropical clade 1, closely related to G1842 from Zambia, with strong support (99% SH-aLRT values, 100% ML ultrafast bootstrap).

### Taxonomy

#### *Inosperma afromelliolens* Eyssart. & Buyck, Cryptog. Mycol. 42(5): 69 (2021), Figs 2, 3 and 6F

*Description*. **Pileus** was 7–25 mm in diameter, conical, umbo, plane to convex, surface slightly rimose, dry, radially fibrillose, generally smooth with some fissured margins, honey-yellowish (3A2), with no color change on bruising or cutting**. Lamellae** 1 mm deep, entire, sub-horizontal, white, with slightly flocculose edges, pale smooth, and obtuse at the margin. **Stipe**:19–35 × 1–4 mm in diameter; white, cylindrical, central, uniform, slightly enlarged at the base, fibrillose, base slightly bulbous, flesh white. **Odor** and **taste** were not distinctive. **Basidiospores** were smooth, ellipsoid to cylindrical, pale yellow, (8) 8.3–**10.3**–12 (12.2) × (4) 4.3–**5.1**–6 (6.1) μm, Q = (1.5) 1.6–**2.0**–2.4 (2.5). **Basidia** 28–40 × 7–12 μm, clavate, 2–4 sterigmates. **Cheilocystidia** 35–42 × 8–15 μm, cylindrical to clavate, thin-walled, and hyaline. **Pleurocystidia** absent. **Pileipellis** a cutis with cylindrical, smooth, thin-walled hyphae, 3–7 μm diam. **Stipitipellis** a cutis, regularly arranged, hyphae 5–10 μm diam., parallel, filamentous. **Caulocystidia** 20–40 × 7–9 μm diam, piriform, sometimes utriform, observed at the top of the stipe. **Clamp connections** were common in all tissues.

**Fig 2:**
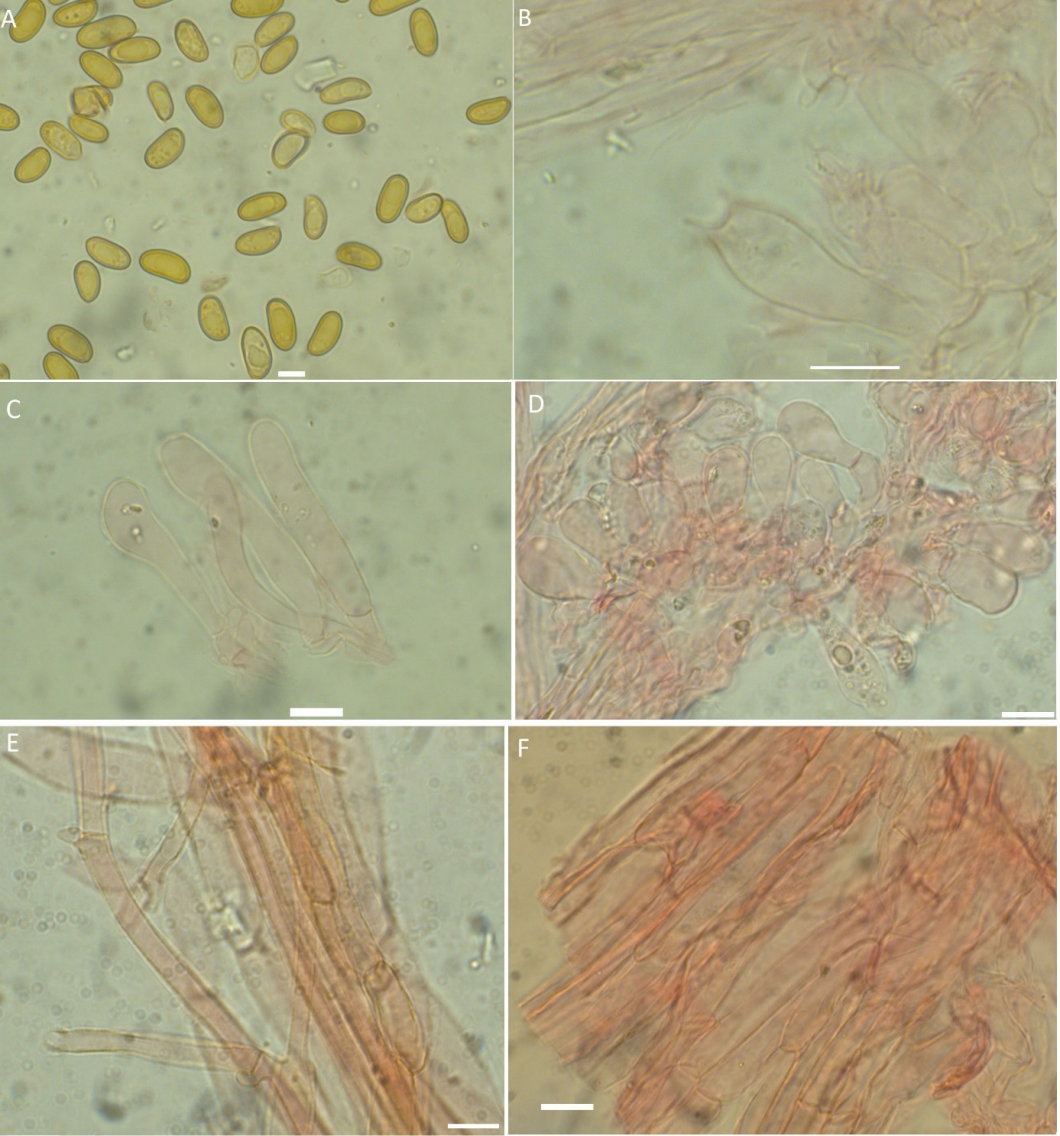
A–F. *Inosperma afromelliolens* (HLA0754), microscopical characters in KOH and Congo Red. A. Basidiospores, B. Basidia, C. Cheilocystidia, D. Caulocystidia, E. Pileipellis F. Stipitipellis. Scale bars: 5 μm (A); 10 μm (B–F).

**Fig 3:**
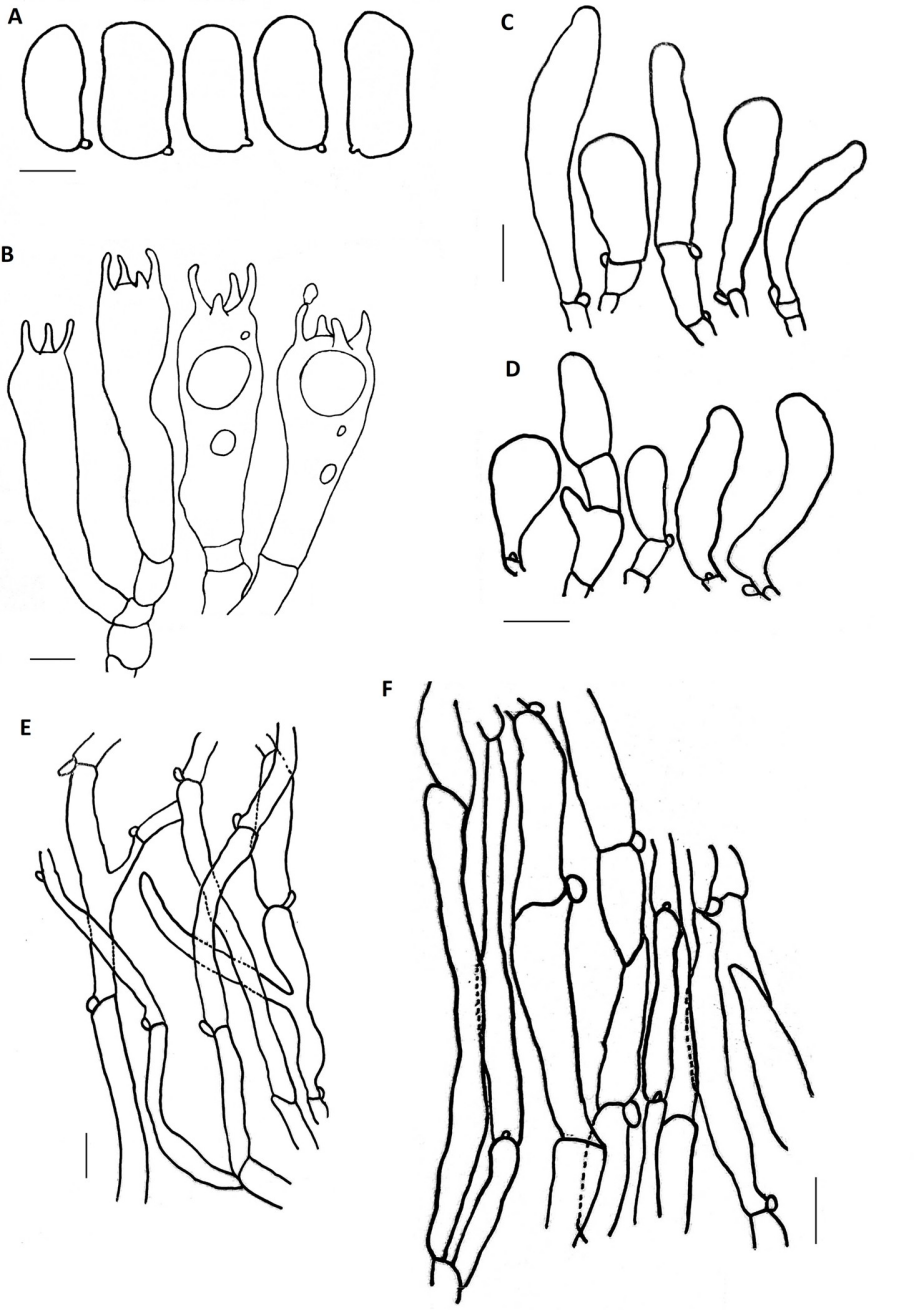
A–F. *Inosperma afromelliolens* (HLA0754). A. Basidiospores B. Basidia, C. Cheilocystidia, D. Caulocystidia, E. Pileipellis F. Stipitipellis. Scale bars: 5 μm (A-B); 10 μm (C–F).

*Habitat*. Woodlands dominated by *Isoberlinia doka and I*. *tomentosa*. Occurrences from June to September.

*Specimens examined*. **Benin**, **Borgou Province**, N’dali Region, in Forest Reserve of N’dali, 8.5456667°N, 2.8403333°E, on soil in woodlands dominated by *I*. *doka*, 04 July 2017, Leg. Aïgnon HL., Voucher (HLA0407); GenBank accession: *ITS* (**MT534296**) and *LSU* (**MT560736**); ibid. 01 September 2017, Leg. Aïgnon HL., Voucher (HLA0468); GenBank accession: *ITS* (**MN096191**), *LSU* (**MN097883**), and *RPB2* (**MN200774**); ibid., voucher (HLA0469); GenBank accession: ITS (**MT534294**) and LSU (**MT560738**/ **MT536757**); ibid. Tchaourou Region:92546667°N, 27230000°E, on soil in the forest of Okpara in a woodland dominated by *I*. *doka*, June 7, 2017, leg. Aïgnon HL., voucher (HLA0355) GenBank accession: *ITS* (**MT534291**); ibid., June 30, 2017, leg. Aïgnon HL., Voucher (HLA0405); GenBank accession: *ITS* (**MT534292**) and *LSU* (**MT560737**); ibid. 9.2446277°N; 2.7262333°E on soil in Okpara forest in a woodland dominated by *I*. *doka*, 07 July 2021, leg. HL. Aïgnon, a voucher specimen (HLA0754), was deposited at UNIPAR. GenBank accession numbers: *ITS* (**OQ300372**), *LSU* (**OQ300369**), *RPB2* (**OQ435246**); *TEF1* (**OQ441164**).

The voucher specimens (HLA0355, HLA0405, HLA0407, HLA0468, HLA0469, and HLA0754) examined in West Africa were morphologically, anatomically, and phylogenetically close to *I*. *afromelliolens* (Table 2).

**Table 2:** Comparative diagnostic features between the original description of *Inosperma afromelliolens* and its collections from West Africa.

*Inosperma afromelliolens*	PC0088778 (Type)	Voucher (HLA0754)
**Pileus**	10–30 mm	7–25 mm
**Lamellae**	Sub-horizontal, slightly flocculose edges, obtuse at the margin, broad, dull beige then slightly ochraceous, rather brownish, (1.5) 2–3 mm	Sub-horizontal, white, bulbous, with slightly flocculose edges, pale smooth, obtuse at the margin, 1 mm
**Stipe**	(20) 25–40 × 3–4 (5) mm	19–35 × 1–4 mm
**Context color**	Whitish	Whitish
**Spore size (ìm)**	(7.5) 8–9 (10) × 4–4.5 (5.5) μm	(8) 8.3–12.2 (12) x (4) 4.3–6 (6) μm
**Basidia**	Clavate, 4-spored, (20) 22–26 (28) × 7–8 μm	Clavate, 2–4 spored 28–40 × 7–12 μm
**Cheilocystidia**	Broadly clavate, sometimes subutriform, (15) 20–40 (45) × (8) 10–13 (15) μm	clavate, cylindrical to thin-walled, hyaline, 35–42 × 8–15 μm
**Host plants**	In miombo woodland	Woodlands dominated by *Isoberlinia doka* and *I*. *tomentosa*

#### *Inosperma macrocarpa* Aïgnon & Yorou sp. nov

*MycoBank No*. MB 846514

Syn. *Inocybe gbadjii* De Kesel *nomen provisorum*, in Boa, Wild Edible Fungi, A global overview of their use and importance to people (Rome): 104 (2004) (nom. inval., art. 39.1(a) ‐ Shenzhen).

**Fig 4:**
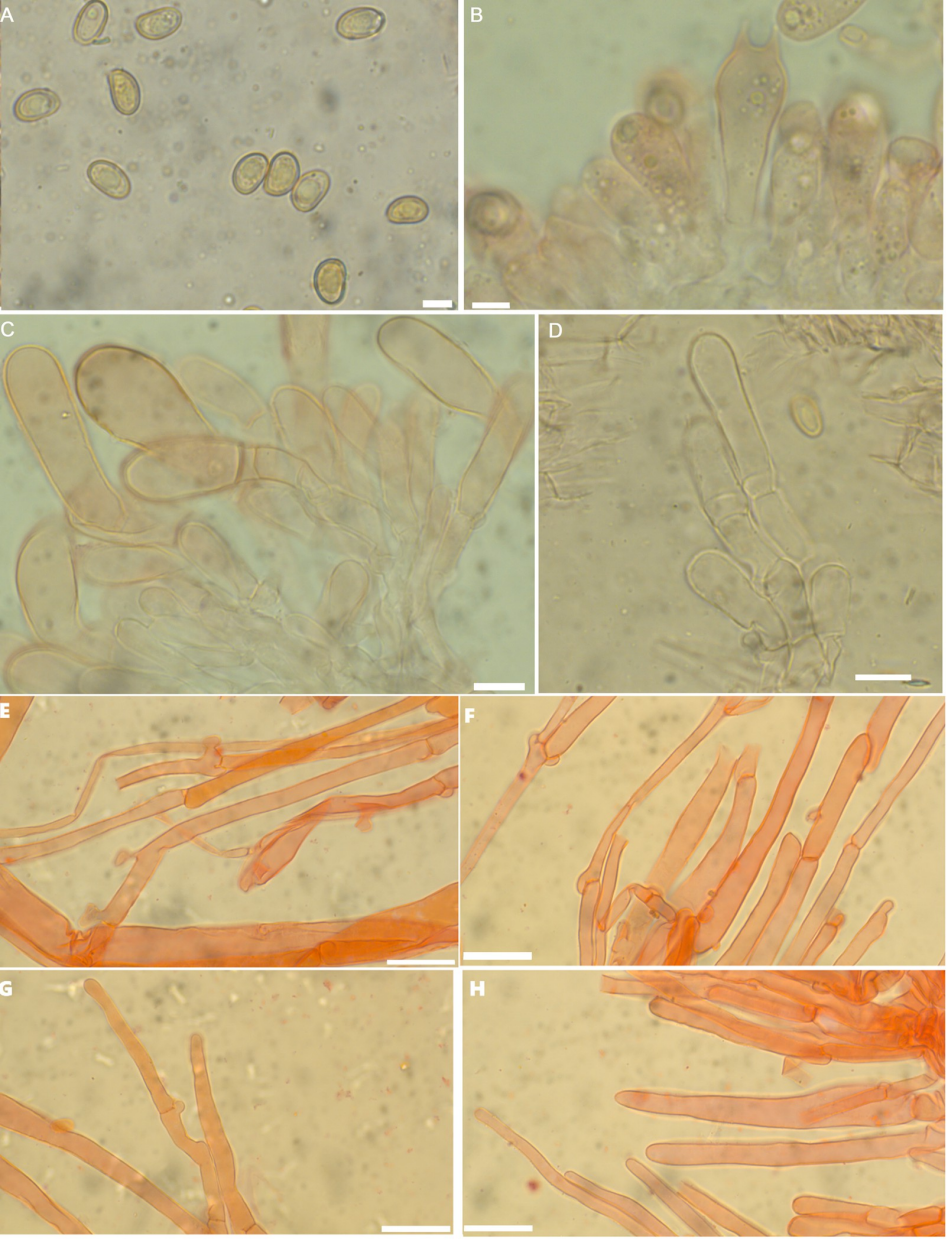
A–H. *Inosperma macrocarpa* (HLA0791), microscopical characters in KOH and Congo Red. A. Basidiospores B. Basidia, C. Cheilocystidia, D. Caulocystidia, E. Pileipellis F. Stipitipellis, G. Pileipellis hyphae H. Stipitipellis hyphae. Scale bars: A–B = 5 μm, C–D = 10 μm, E–H = 20 μm.

**Fig 5:**
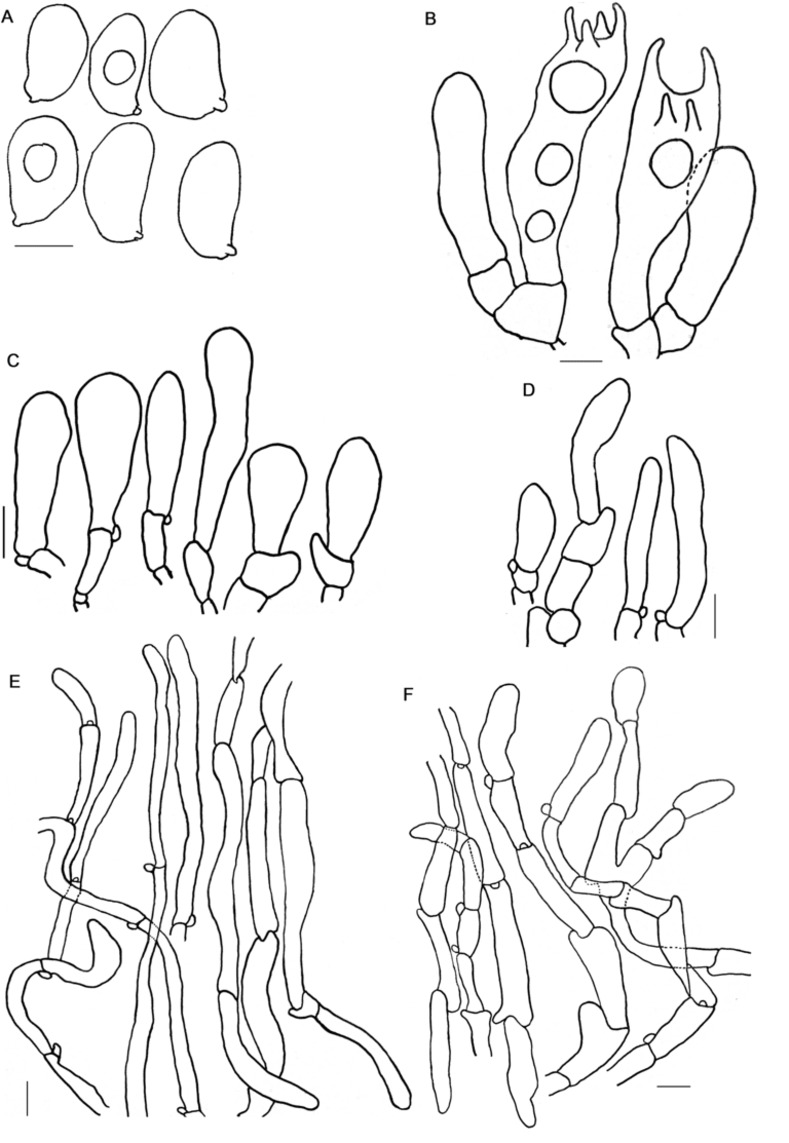
A–F. *Inosperma macrocarpa* (HLA0791). A. Basidiospores B. Basidia, C. Cheilocystidia, D. Caulocystidia, E. Pileipellis F. Stipitipellis. Scale bars: A–B = 5 μm, C–F = 10 μm.

**Fig 6:**
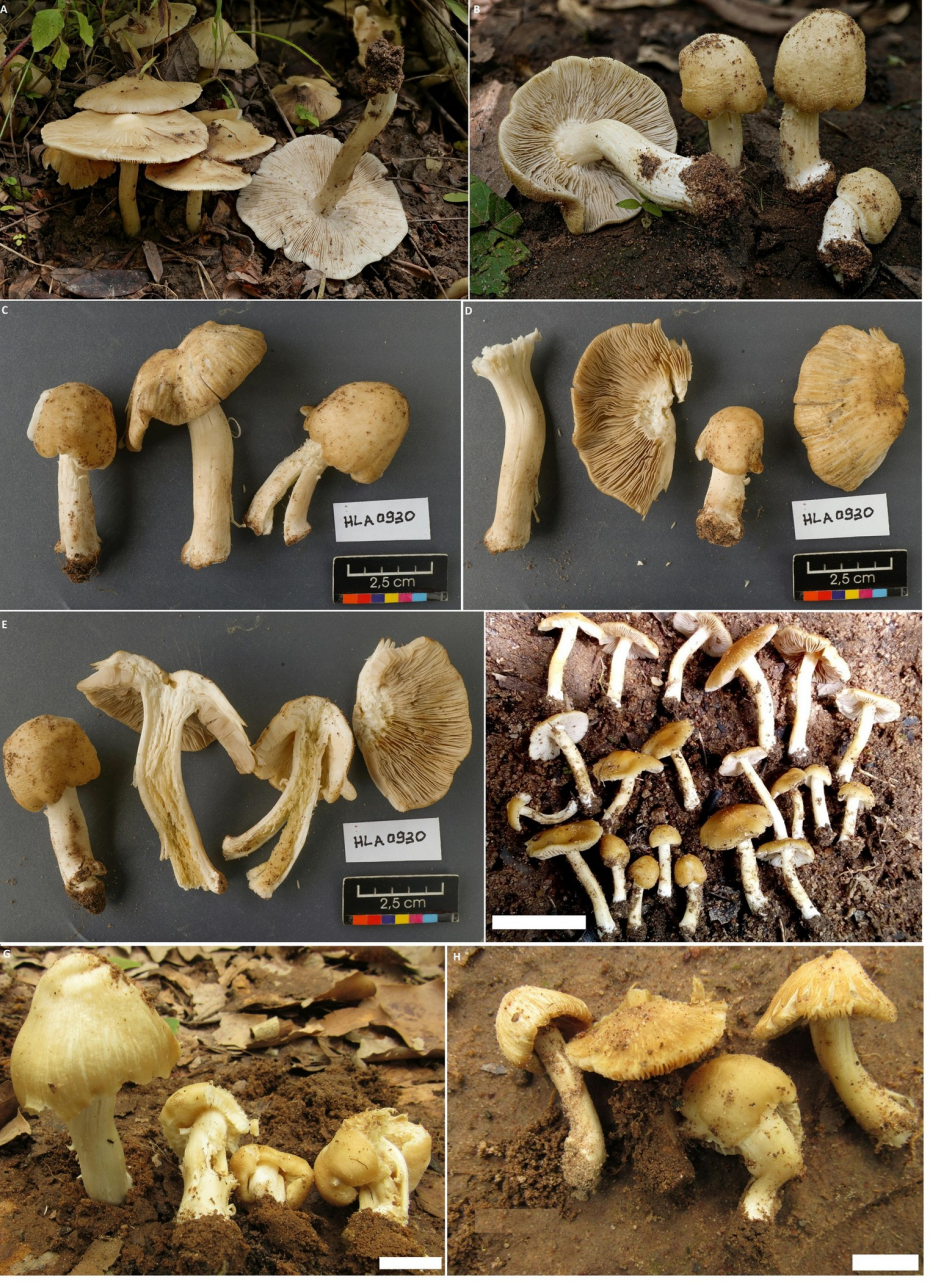
Macromorphology of: A–E, G–H. *Inosperma macrocarpa* (A–E = HLA0930, G = HLA0791, H = HLA0920) and F. *Inosperma afromelliolens* (HLA0407). Scale bar: 2.5 cm (A–F) and 1 cm (G–H).

Figs 4, 5 and 6A–6E, 6G and 6H

*Diagnosis*. *Inosperma macrocarpa* differs from all *Inosperma* species known from tropical Africa by its larger basidiomata (37–86 mm), plano-convex to convex, and slightly conical pileus.

*Holotype*. **Benin**, **Atacora Province**, Boukombé Region, Koussoucoingou, 10.173066°N, 1.196233°E, in a gallery forest dominated by *Uapaca guineensis* and *Berlinia grandiflora*, September 16, 2021, Leg. Aïgnon HL., Voucher (HLA0791 deposited at UNIPAR) GenBank accession numbers: *ITS* (**OQ300373**), *LSU* (**OQ300370**), *RPB2* (**OQ435244**), and *TEF1* (**OQ441169**).

*Etymology*. *macrocarpa* (L.) refers to large basidiocarps.

*Description*. **Pileus** was 37–86 mm in diameter, wide, plano-convex to convex, slightly conical with age, yellowish white (3A2) at the margin, dark yellowish white in the middle (4A2), no color change on bruising or cutting, surface wavy, margin incurved, streaked, flesh uniform, surface dry, and glabrous to fibrillose. **Lamellae** 2 mm deep, crowded, adnexed, 30 reaching stipe, with some tiers of yellow-brown lamellula (5B5). **Stipe** 49–65 mm high, 8–12 mm in diameter, white, cylindrical, central, equal, straight, sometimes entirely fibrillose at the top, inseparable from the cap, sometimes curved, base slightly bulbous to marginally bulbous, flesh white to yellowish (3A2). **Stipitipellis** was glabrous to fibrous. **Odor** and **taste** were initially neutral, then reminiscent of almonds. **Basidiospores** (6.6) 8.0–**9.8**–10.5 (12) × (3.6) 5.0–**6.2**–6.6 (8) μm, Q = (1.2) 1.3–**1.6**–2.0 (2.2), smooth, ellipsoid. **Basidia** 24–35 × 7–12 μm, clavate, 2–4 sterigmates. **Cheilocystidia** 26–35 × 6–15 μm diam, clavate to piriform, sometimes subutriform. **Pleurocystidia** absent. **Pileipellis** a cutis of cylindrical hyphae, 5–8 μm broad, filamentous with incrusting pigment. **Stipitipellis** a cutis, regularly arranged with subparallel hyphae 4–15 μm diam, septate, filamentous, no reaction with KOH. **Caulocystidia** 15–33 × 6–11 μm diam, utriform, observed on the upper third of the stipe. **Clamp connections** were common in all tissues.

*Habit*. In small or large groups, scattered on soil.

*Habitat*. Woodland forest dominated by *Isoberlinia doka* and/or *I*. *tomentosa* and *Uapaca togoensis* and gallery forest dominated by *Uapaca guineensis* and/or *Berlinia grandiflora*. Occurrences from June to September.

*Geographical distribution*. Currently known: Benin

*Edibility*. The new species is used for consumption in Borgou Province with the local name osousou kaka in the Nagot language and Zou province with the local name kocholé in the Fon language.

*Additional specimens examined*. **Benin**, **Atacora Province**, Boukombé Region, in the gallery forest of Koussoucoingou, 10.176230°N–1.203339°E, on soil in woodlands dominated by gallery forests with *Uapaca guineensis* and *Berlinia grandiflora*, September 15, 2021, Leg. Aïgnon HL., voucher (HLA0787), GenBank accession numbers: *ITS* (**OQ300390**), *LSU* (**OQ286290**), *RPB2* (**OQ427873**), and *TEF1* (**OQ441166**); ibid., 10.175111°N, 1.202631°E, leg. Aïgnon HL., voucher (HLA0788), GenBank accession numbers: *ITS* (**OQ300391**), *LSU* (**OQ286291**), *RPB2* (**OQ435242**), and *TEF1* (**OQ441167**); ibid., Leg. Aïgnon HL., voucher (HLA0790), GenBank accession numbers: *ITS* (**OQ300392**), *LSU* (**OQ286292**), *RPB2* (**OQ435243**), and *TEF1* (**OQ441168**); ibid., 10.174022°N, 1.203330°E, Leg. Aïgnon HL., Voucher (HLA0790), GenBank accession: *ITS* (**OQ300392**), *LSU* (**OQ286292**), *RPB2* (**OQ435243**), and *TEF1* (**OQ441168**); ibid., 16 September 2021, Leg. Aïgnon HL., voucher (HLA0790), GenBank accession numbers: *ITS* (**OQ300392**), *LSU* (**OQ286292**), *RPB2* (**OQ435243**), and *TEF1* (**OQ441168**); ibid., Leg. Aïgnon HL., voucher (HLA0792), GenBank accessions: *ITS* (**OQ300393**), *LSU* (**OQ286293**), *RPB2* (**OQ435245**), and *TEF1* (**OQ441170**); ibid., **Borgou Province**, Tchaourou region, Okpara forest, 9.2446277°N, 2.7262333°E, on soil in woodlands dominated by *Isoberlina doka*, August 18, 2022, leg. HL. Aïgnon, specimen voucher (HLA0917); ibid., leg. HL. Aïgnon, specimen voucher (HLA0920); ibid., August 22, 2017, leg. HL. Aïgnon, specimen voucher (HLA0456); ibid., Wari Maro, Forêt classée de Wari Maro, 9.164733°N, 2.159917°E, on soil in woodlands dominated by *Isoberlina doka* and *Uapaca togoensis*, June 20, 1998, leg. A. De Kesel, voucher (ADK2166, deposited at BR, BR 5020112676592); ibid., **Zou Province**, Savalou, near Ouèssè, Reserve forest of Gbadji, 7.952167°N, 1.967867°E, on soil close to an inselberg, in a woodland dominated by *Isoberlina doka* and *Uapaca togoensis*, June 19, 1997, leg. A. De Kesel, voucher (ADK2618, deposited at BR 5020115701772).

Reprinted from [AIGNON] under a CCBY license with permission from [AIGNON], original copyright [2022].

### Toxin detection

The weight of the tested samples was 0.01236 ± 0.009 (Table 3). After comparing the retention time (0.89 min) and relative deviation (5.11%) with standard muscarine in the allowance of ± 25% relative range, muscarine was not detected in the four samples of *I*. *macrocarpa* and the one sample of *I*. *afromelliolens*. The calibration curve for muscarine generated during validation was *y* = 16702.81879*x* + 4.18331e^4^ (*r* = 0.99505) for muscarine concentrations in the range 2–100 ng/mL (*y* = peak area, and *x* is = muscarine concentration, *r* = correlation coefficient).

**Table 3:** Weights, extraction solution volume, and muscarine contents of tested samples.

Species	Voucher	Weight (g)	Methanol-water (5:95, v/v) (ml)	Muscarine
***Inosperma afromelliolens***	HLA0754	0.0025	2	n.d.
***Inosperma macrocarpa***	HLA0787	0.0071	2	n.d.
***Inosperma macrocarpa***	HLA0790	0.0100	2	n.d.
***Inosperma macrocarpa***	HLA0792	0.0164	2	n.d.
***Inosperma macrocarpa***	HLA0788	0.0258	2	n.d.

“n.d. indicates not detected.

## Discussion

Here, we present *Inosperma macrocarpa* as a novel species. Based on the morphological and molecular similarities between the collections from West Africa and *Inosperma afromelliolens* collected elsewhere, to date only known in Zambia, our data indicate that the distribution range of *I*. *afromelliolens* is broader than previously reported. Based on their morphological characteristics, collections from West Africa (voucher specimens HLA0468, HLA0469, and HLA0355. HLA0405 and HLA0754) were similar in size (7–25 mm versus 10–30 mm), color, and anatomical features to the collections of *I*. *afromelliolens* (voucher PC0088778) (Table 3). This is in agreement with our phylogenetic analysis, which did not show any differences between these collections (Fig 1).

*Inosperma macrocarpa* presents morphological characteristics of taxa from the genus *Inosperma*, especially a radially rimose, fibrillose, or squamulose pileus and the absence of pleurocystidia [1]. Molecular analysis based on the combined data of *ITS*, *LSU*, *RPB2*, and *TEF1*, confirms its position in *Inosperma*. *I*. *macrocarpa* is nested in Old World Tropical clade 1 and is close to the undescribed collections of *Inosperma* sp. G1842, and *Inosperma* sp. BB3233 from Zambia, with weak (57%) SH-aLRT values and strong (98%) ML Ultrafast bootstrap support. Morphologically, *I*. *macrocarpa* is close to *I*. *cookei* a European species; however, in terms of pileus size (37–86 mm), *I*. *macrocarpa* is closest to *I*. *erubescens*.

The *Inocybaceae* have many muscarine species, but the genus *Inosperma* has a few toxic species [5, 8, 9] that have been systematically reported in incidents of mushroom poisoning in tropical Asia [7, 10, 11]. In Africa, poisoning due to the consumption of wild mushrooms is often not reported and is difficult to assess [49]. In particular, no cases of mushroom poisoning have been officially reported in Benin, although mushrooms from families that include toxic species, such as *Inocybaceae* are consumed in this region. The first published records of *Inosperma macrocarpa* date back to twenty years back [50, 51]. These records, provisionally named *Inocybe* sp. and *Inocybe gbadjii* (ADK2166 and ADK2618, respectively), indicate that this taxon is consumed and appreciated by the local people. This prompted us to perform a toxicity analysis, which revealed a negative result for muscarine content in these taxa as well as in *I*. *afromelliolens* (Table 2).

This study is the first to focus on the toxicity of *Inosperma* species in Africa and increase the diversity of taxa in *Inosperma* to eight species, five of which are distributed in West Africa: *I*. *africanum*, *I*. *bulbomarginatum*, *I*. *flavobrunneum* [4], *I*. *afromelliolens* [52], and *I*. *macrocarpa*. *Inosperma afromelliolens* is widely distributed and present in East Africa in Zambia, along with *I*. *boeticum*, *I*. *submaculatum* [52], and *I*. *misakaense* [53]. Our data suggest that the diversity of *Inosperma* in Tropical Africa is greater than the currently known, and many species remain to be identified.

## Supporting information

S1 Text (TXT)
